# Abstracts and Gleanings

**Published:** 1880-09-20

**Authors:** 


### ABSTRACTS AND GLEANINGS.

**Dr. Tanner's Fast.**—The fast commenced on June 28th 1880. The following table exhibits the temperature, pulse, respiration, etc., during the forty days.

| *Day.* | *Weight.* | *Highest Temperature.* | *Highest Pulse* | *Respiration.* | *Ounces of Water Voided* | *Ounces of Water Drank* |
|---|---|---|---|---|---|---|
| First | 157½ | 99 | 88 | 16 | 21 | 48 |
| Second | — | *98¾ | 84 | 16 | 8½ | 24½ |
| Third | 153 | 98½ | 78 | 16 | 22½ | — |
| Fourth | — | 98½ | 84 | — | 19½ | — |
| Fifth | 147½ | †98⅞ | 100 | — | 17¼ | — |
| Sixth | — | 98¾ | 100 | — | 14 | — |
| Seventh | 143 | 98½ | 90 | 16 | 14 | — |
| Eighth | — | 98½ | 84 | 14 | 13 | — |
| Ninth | 141¾ | ‡98¼ | 116 | 16 | 13 | — |
| Tenth | — | *99¼ | 98 | 14 | 13¾ | 4 |
| Eleventh | 139¾ | 100 | 94 | — | 16 | — |
| Twelfth | — | *99¼ | 93 | 14 | 14 | — |
| Thirteenth | 136¼ | 98 | 100 | 16 | 15½ | — |
| Fourteenth | 133 | *98¾ | 96 | 16 | 14 | — |
| Fifteenth | — | 98 | 107 | — | 17 | 1 |
| Sixteenth | — | 98 | 108 | 14 | 18¾ | 39 |
| Seventeenth | 137½ | 99 | 98 | 16 | 33½ | 66 |
| Eighteenth | 136½ | *98¾ | 76 | 16 | 36¾ | 51 |
| Nineteenth | 136 | 99 | 84 | 15 | 26½ | 46½ |
| Twentieth | 135 | 99 | 82 | 15 | 45 | 39½ |
| Twenty-first | 135 | 99 | 84 | 16 | 33 | 51 |
| Twenty-second | 134 | 99 | 72 | 15 | 29 | 18½ |
| Twenty-third | 133½ | 98 | 88 | 18 | 30½ | 23½ |
| Twenty-fourth | 132 | *99¼ | 84 | 16 | 21½ | 30 |
| Twenty-fifth | 131½ | *98¾ | 72 | 16 | 19¼ | — |
| Twenty-sixth | 131½ | *98¾ | 80 | 16 | 19½ | 12½ |
| Twenty-seventh | — | *98¾ | 76 | 16 | 12 | 8 |
| Twenty-eighth | 129¾ | *98¾ | 72 | 16 | 9¼ | 19 |
| Twenty-ninth | — | *98½ | 74 | 16 | 22¼ | 14 |
| Thirtieth | 130 | *98¾ | 84 | 14 | 28 | 10 |
| Thirty-first | 128 | *98½ | 74 | — | 9 | 14 |
| Thirty-second | 127½ | *98¾ | 72 | 15 | 14 | 9 |
| Thirty-third | 126½ | 99 | 78 | 9½ | 5½ | 15 |
| Thirty-fourth | 126½ | 99¼ | 78 | 14 | 12½ | 7 |
| Thirty-fifth | — | 98 | 78 | — | 10 | 17 |
| Thirty-sixth | — | — | 74 | — | 9¾ | 19¼ |
| Thirty-seventh | 125½ | 98¾ | 74 | 14 | 27½ | 12½ |
| Thirty-eighth | — | 99¼ | 78 | 15 | 13 | 8 |
| Thirty-ninth | 122½ | — | — | 13 | — | 3 |

*Whenever the asterisk appears it indicates that for convenience in typography there is a change from a fifth in the original manuscript to a quarter in print, or three-fifths in manuscript to three-quarters in type. Where the † is the original manuscript gives the temperature as ninety-eight and nine-tenths, and for convenience in typography it is made in print 98⅞. ‡ This should be ninety-eight and three-tenths.

—*N. Y. Med. and Surg. Journal*

**The Effects of Conception on the Nervous System.**—March 2d., I was called to see Mrs. M., aged 21 years; married six months; very robust and of a sanguineous temperament. Since puberty she has menstruated regularly and without any discomfort or nervous symptoms, up to the present date, which is her catamenial period, but it has failed to appear. She has heretofore enjoyed perfect health. With this history the following symptoms presented: A slight, whitish fur on the tongue; pulse a little excited; respiration somewhat hurried and forced; no symptoms of malarial poison; no hysteria; urine tested and found normal; bowels open; disposed to sleep all the while, and while asleep would pull and pick violently at the bedclothes and indulge in low mutterings; when aroused her mind would become perfectly clear and calm; complained only of a severe pain in the lower part of her spine; unable to move herself in bed without assistance; could neither stand nor sit alone; if she attempted to walk her toes would drag on the floor; she would pass into a sleepy stupor while standing up, and would say her sleep was natural; complained of no pain in head; no loss or increase of sensation. A vaginal examination showed a softened condition about the os uteri; no dilatation. The most attractive and unusual symptom of great nervous irritation was the rapid contraction and dilatation of the pupils. They would contract and dilate to their full extent about six or eight times to the minute, entirely uninfluenced by light, and seeming to affect the vision in no way in either state.

I will state that these symptoms, so far as I could learn, had developed the night previous, as she was up and attending to her duties the day before I saw her, complaining only of a backache.

From the foregoing history and symptoms I diagnosed the case as one of hyperæmia of the brain and spinal cord, caused by this, her first, conception, producing a reflex action. With this conviction I proceeded to apply wet cups along the spine, and removed several ounces of blood. I also ordered a preparation of bromide of potassium and valerian and left the patient, expressing the hope that the system would accustom itself to the physiological functions going on, and that these symptoms would soon pass away.

Two days later I repeated my visit and found her condition no better, except that the cupping had partially relieved the pain in the spine. I applied the cups again and drew off more blood. To open the bowels, ordered large doses of calomel and ipecacuanha.

Saw the patient next day. Pain over the spine greatly relieved; not so much paralysis; no abatement of other symptoms; cathartic acted freely. In this condition she remained for nearly two months, the symptoms gradually increasing in severity and yielding to no treatment that I could institute.

April 29th. Found her in slight convulsions, gradually increasing in severity and frequency. I advised the induction of abortion.

April 30th. With the assistance of my friend Dr. John Rainey, who had seen the case and concurred with me in the mode of procedure, I introduced a probe into the uterine cavity and broke up the membranes. She bore the operation well, and aborted in about thirty hours, throwing off a fœtus I supposed to be six or eight weeks old. There was no sudden reaction, but her symptoms gradually became milder, and I can pronounce her, May 20th, entirely recovered.

In the treatment of this case there arose an immaterial, though interesting, question in my mind. It was, whether this condition which, toward its close so greatly endangered her life, arose from the suspension of a previous regular physiological act, or from the taking on of a new physiological condition.

I think it arose entirely from the conception, for the suspension of menses was no less physiological than the conception. The point that created this question was this: She menstruated the last time about February 1st, and conception took place a few days later, judging from the subsequent non-appearance of the catamenia and the size of the fœtus. Now, she carried that fœtus, without discomfort, from about February 5th to March 1st, which was her time, and then the foregoing symptoms set up. This is not so strange when we reflect and see that the uterus, even after conception, often takes on its regular monthly congestion, and in some instances the flow comes on.

Having presented this case in detail, I shall not discuss it further than to say I think it too often the custom of physicians to postpone or refuse such timely interference, in similar cases, as is necessary for the safety of the mother. The danger of the operation in this case was enhanced, because of my postponing until convulsions came on.

I humbly offer the report of this case to the profession, for such consideration and discussion as the rarity of its symptoms and obscurity of its pathology may demand.—DR. P. W. JOHNS, *in Med. and Surgical Reporter.*

**Leucocythæmia.**—Gentlemen, we have here a young girl, eighteen years old, whose mother tells us that she was quite well until four months ago. Four months ago her mother noticed that the glands in her neck were becoming swollen. This increase in size of the glands has apparently gone on very rapidly since that time, for now we find a very considerable number of the glands enlarged, the glands affected being principally the glands of the neck, the glands running down from the neck into the thoracic cavity and the axillary glands. The inguinal glands she says are not involved. The tonsils are a little involved, not very much.

The girl's color, as you will observe, is still very good, and she apparently complains of nothing except this enlargement of the glands. The mother states that the girl has not been unwell for four months; that is, that her menses have not come on the past four months.

That makes the whole of the case then. A girl having no symptoms except the cessation of the menses, and presenting herself with a number of enlarged glands as you see. I should say also that the spleen is enlarged. Now, what is the matter with her? "Leucocythæmia." Yes, probably. Of course, we cannot be certain of that without examining the blood. It is possible for a person to have such enlarged glands as this girl has, and have an enlarged spleen also, and yet not leucocythæmia. There may be no increase of the white blood globules at all. What would the patient be suffering from then? If we examine this girl's blood and do not find any increase of the white blood globules—"Anæmia." Well, simple anæmia would not account for the enlargement of the glands. "Struma." Well, that does not mean much, doctor. There are two conditions with which we meet which are alike except as regards the condition of the blood. We have the same enlargement of the glands with or without enlargement of the spleen in both cases. But in one set of cases there is an increase of the white blood globules. This we call leucocythæmia or leucæmia. In the other state the glandular changes are the same, but there is no increase of the white blood globules. Now, what is it called? "Leucocytosis." No, the word leucocytosis is applied to cases in which there is a slight increase of white blood globules—cases which are not fairly cases of leucocythæmia, and in which there is a little increase of the white blood globules. But we are supposing that when we come to examine the blood we will find no increase of the white blood globules. We cannot tell yet whether there is or is not. If there is, then the case is one of leucocythæmia; if there is not, then what is the trouble? There is a disease which is usually called pseudo-leucæmia, or false leucæmia; that is, a disease resembling leucæmia or leucocythæmia (for those two words are used in the same sense), but yet without the characteristic change in the blood. Those two conditions, leucæmia and pseudo-leucæmia are very closely allied indeed. The cases run very much in the same course; they present very much the same symptoms, and really the only apparent difference between them relates to the condition of the blood.

In either case the treatment is the same. If the patients at the time you see them are not anæmic, then you direct your attention to the reduction of the size of the glands. If they are anæmic they may be so either with or without an increase of the white blood globules. You observe that by anæmia we do not mean an increase of the white blood globules. We mean a decrease of the red ones, and a patient may come to you like this girl, with enlarged glands, and she may be anæmic or not. In this case she is not. The color is still good. In this particular case then the duration being short, and the condition of the blood, so far as the red blood globules is concerned, being still good, we shall direct our attention directly to the condition of the glands.

Now, there is one drug which has special power in diminishing the size of the lymphatic glands if they are enlarged, and that drug is iodine. And for this particular purpose, if you want to get the real effects of the iodine, the best thing you can do is to give it in the form of Lugol's solution—of Lugol's solution of iodine. And you will give the girl, to begin with, five drops of this—five drops taken in water three times a day, and taken after meals. The only objection to this medicine is that some stomachs will not bear it. It makes some persons so sick at the stomach that we are unable to give it to them, but I imagine that this girl will be able to take it. That will be the first thing for her to do. Begin the treatment then with Lugol's solution in five-drop doses three times a day; continue that for a week. Then the dose should be gradually increased by a drop at a time until she shall take ten drops three times a day. That is one plan for treating these cases, if you see them in the condition in which this girl is, the general condition being still good.

Another plan is to get them under the constitutional effect of arsenic. You give them arsenic in increasing doses until you have reached a point of tolerance. Then you stop the arsenic for a time, and allow the patient to recover from its effects. Then you can repeat this again. This plan also has considerable effect in diminishing the size of the glands, but generally speaking I think the iodine treatment is to be preferred. If, however, the patient was anæmic at the time you saw her; if we were to see this girl a year from now, she having been left to herself, and should find the glands larger than they are now, and we should find that, instead of having the color which she has now, she should be very pale: she would be anæmic. Then we could do nothing with our iodine. Then we should have to put her upon the use of iron and oxygen. We would treat her as we would any other case of anæmia. DR. DELAFIELD *in Clinic, Detroit Lancet.*

**Consumption on the Salisbury Plan.**—Some time ago I gave the leading ideas of the Salisbury plan in this Journal. For the benefit of those who did not peruse that paper, the following concise statement is introduced. This will enable the student to take in the significance of the actual cases presented.

Dr. Salisbury regards consumption to be a diseased condition or state that is at first systemic, then local, found in the blood one year generally, before the organic pulmonal disease. This is the pretubercular state. The Salisbury plan diagnosticates this state by the addition of a new physical sign. The prime element of the disease is defective alimentation, by which a vegetation is introduced into the blood through the alimentary canal. This vegetation is a yeast plant.

Tubercle is an accident or secondary condition due to capillary embolism.

This embolism is caused by the presence of the yeast vegetation, by the massive fibroid filaments and by the enlarged massal white corpuscles aggregating together. These abnormal form elements make up an essential part of the Salisbury morphology of consumptive blood.

There is then something tangible to the sense of sight in the causation of consumption. It has been photographed by the writer.

The treatment on the Salisbury plan consists in the removal of the yeast from the blood by starving out the yeast. This is done first by regulating the food and giving appropriate medicine. The studies on which this plan is based, extend over a period of twenty-five years and embrace thousands of cases.

The evidence of this statement is as follows: In 1858 Dr. Salisbury had ready for the press, a work in which he describes his experiments with over 2,000 swine. They were fed in various ways, but 1,026 were fed on food filled with yeast.

In ten weeks time 246 swine died; 104 of them were examined after death, and all were found to have in the lungs the disease known as consumption. The writer has perused the records of these *post-mortem.*

At about the same time, Dr. Salisbury made bread with flour raised with the yeast found in the diarrhœal dejections of a third stage consumptive. In 1878 I repeated this experiment and sent a sample of the bread to the editor of this Journal. Per contra, Dr. Salisbury examined one hundred well swine that had been fed on good, sound corn, and found hardly a trace of tubercle in their lungs. Again, Dr. Salisbury took healthy men—hired by the day,—and fed them as he fed the thousand hogs. In every case consumption of the bowels was induced in from ten to fifteen days.

Can the candid and unprejudiced student in medicine ask for more? Here he presented a plan that embraces the cause or rather the synthesis of the disease in animals, verified by *post-mortem* examination. The morphology of the blood is photographed. The reduction in size of the enlarged white blood corpuscles to a normal state, the dissipation of the spores and spore collests, and the restoration or the red discs towards a normal condition, have been photographed and are on record; indeed, the demonstration is made daily. To crown all, cases are cured and the profession is asked to examine them by Dr. Salisbury.

Can anything more be done to present the matter? Yes, there can.

Dr. Salisbury, in connection with myself, offers to go to New York and treat twenty-five cases of consumption sent by intelligent physicians of good character, with a written diagnosis of the case so that there shall be no disagreement afterwards. The physicians to watch the cases through the treatment for three months. If possible arrangements should be made to take photographs of the blood from time to time as records of the progress of the cases, and at the expiration of a year the physicians shall be invited to meet in counsel and judge on the results by the histories.

In this way it is thought that it can be decided best, if the experience of Dr. Salisbury and myself can be realized under other conditions where the profession can watch the progress for themselves, and where the treatment shall be fully and carefully carried out without let or hindrance. The full text of the treatment may be found in the September 1879 No. of the Virginia Medical Monthly, Richmond, Va., or the Scientific American.

The full exposition has been ready for the press for some time, and waits to be produced when asked for. Dr. Salisbury is in no haste, but when the writer considers how far inferior are the present unsatisfactory modes of treating this disease and how much less they have to bring forward to their support, he thinks that one-fourth of a century is long enough to wait. As things now go, one-fourth of the very physicians who read this paper will, in all probability, die of consumption, to say nothing of their patients. So then, it seems to the writer that no time should be lost in the proper presentation and demonstration of this plan.

At any rate, after this, I cannot be blamed for not having tried to make known the new information as to the cause and cure of the disease.—Dr. CUTTER, in *Mich. Med. News*.

**Analysis of a Secret Cancer Plaster.**—Dr. Edmund Andrews in the Chicago Medical Review, says:

A person living not a thousand miles southwest of Chicago, treats such patients as he can convince that they have cancers, as follows: He applies a caustic plaster for six hours, producing a white eschar. Removing the plaster he poultices the spot for twelve hours, and then applies the plaster for six hours more.

In this way the cautery is gradually deepened, and the eschar turns black and begins to separate from the living flesh. When he judges the action to be sufficient, he ceases the use of the caustic until the slough drops out, when, if any suspicious points remain, he reapplies the remedy.

The incompleteness of the work, however, is manifest by the large proportion of cases where the disease reappears in the same spot.

Some of his caustic having fallen into my hands, I gave it to Prof. M. P. Hatfield for analysis. The material was in the form of a brown paste, which, on close inspection, consisted of a fine brown powder imperfectly mixed with a transparent or translucent paste containing the caustic. Applied to the raw surface of a cancer, I found it to turn it white and to produce an eschar.

Prof. Hatfield found the active principle to consist simply of chloride of zinc. A faint trace of iron was present as an impurity, and the coloring matter was a vegetable powder.

The quack claims almost miraculous powers for his secret plaster of chloride of zinc, and finds a considerable number of dupes to employ him.

The moral of this tale should be pondered by surgeons. The general horror of the knife is natural, and is so decided that many minds cannot calmly face it, even with the promise of ether. Such patients will calmly endure great pain from a caustic if they can be spared the thought of the cruel steel. A mental pain is as real as a physical one, and harder to bear. Would it not be really humane in properly selected cases to use caustic more than we do, making up for their clumsiness by attention and determined persistence, so as to be thorough in the extirpation?

Esmarch's painless caustic powder might serve in some cases, and chromic acid, used while the patient is under the influence of morphine, would serve a quick turn in others.

Esmarch's painless powder is prepared after the following formula:

| | | |
|---|---|---|
| ℞ | Arsenious acid.................................. | 1 part. |
| | Sulph. morph.................................... | 1 part. |
| | Calomel......................................... | 8 parts. |
| | Pulv. gum Arabic................................ | 48 parts. |

Mix. Sprinkle thick every day on a surface either raw, or denuded of cuticle by a blister.

**The Use of Quinine in Connection with Nervous Sedatives.**—Dr. Gray said: Great relief was to be expected from the bromides in robust patients; but not to the same extent in the case of the weak and anæmic. His own experience had convinced him that there was considerable danger in using them freely in certain instances, and he had met with one case in which a fatal result was thus produced. He was a firm believer in their efficacy in epilepsy, as a general rule; but at the same time he felt that under some circumstances they should be used, if at all, only with extreme caution.

For the past two years he had been in the habit of prescribing quinine in connection with the bromides, and he could but express himself as more than satisfied with the results obtained by this combination. At first he had employed it with timidity and in very small doses, as he feared, from what he had been taught, that it might perhaps interfere with their action, and only aggravate the trouble present; but afterwards he had used it much more freely, and always with very beneficial effects. His practice now was to give first a sufficient quantity of the bromides to produce bromism, and then two or three grains of quinine three times a day in addition. He had met with a few cases in which quinine was not well tolerated, but as a rule such patients were able to stand the full sedation of the bromides; while in some instances he had deemed it advisable to stimulate the system with quinine before commencing the use of the bromides, on account of the weak condition of the patient.

All his experience went to show that quinine actually increased the effect of the bromides, hyoscyamin, and belladonna, and he had also found that all these agents were much better borne by the system, as well as more efficient in their action, when administered in combination with quinine than when the latter was omitted.

The happy effect of the quinine with hyoscyamin was well shown in a case of puerperal mania which he had seen in consultation. When hyoscyamin was given alone it seemed to be of no service; but when quinine was added to it, although there was no malarial complication in the case, its use was followed by the most happy results.

Dr. Jewell stated that he found strychnia exceedingly useful in combatting the depressing effect of the bromides when given in sufficient quantities to control epileptic attacks, and that it did not, as might perhaps at first be supposed, increase reflex irritability, and thus render the patient more liable to fits. He had not had much experience with quinine, however, in this way.—*Amer. Neurological Assoc. Trans.*

**Prostration and Nervousness after Coition.**—Nervousness and sleeplessness and headache and prostration following coition, when performed but rarely, are evidences of some form of sexual exhaustion and are worthy of attention even though there should be no other sign of trouble in that function.

In practice we find, however, that this symptom does not exist alone; it is always a part of a group. In one extraordinary case for which I was consulted, coition as infrequent as once in three months produced unpleasant effects, but the individual thus sensitive could sleep at any time and as long as he wished, and was capable of severe and responsible mental labor.

There are cases not so uncommon as was formerly supposed, whose genital function is in a condition analogous to the extreme forms of nervous dyspepsia—intolerant of any functional activity, no matter how mild or cautious.

There are persons who cannot eat even a crust of bread without experiencing discomfort; just so there are persons who cannot indulge at all in coition without experiencing more or less of the symptoms that are supposed to follow only great excess; this function is a poison to them—the only difference in the evil effects of moderation and excess being of degree only.

Sexual intercourse in the normal man is a sedative and tonic; it induces sleep, quickens the appetite and inspires activity; the slight and pleasing languor that follows it are physiological. In cases of this kind, as I have many times noticed, coitus is followed the next day by an unusual sensitiveness of the urethra, as is observed on introducing the sound or other instruments.

In the time of their appearance these bad effects of sexual excitation are remarkably inconsistent; in some persons, not manifesting themselves for two days, or the second day; in other cases, appearing at once after the act. This same inconsistency obtains in other symptoms and after the exercise of other functions; thus a clergyman who consulted me was troubled not by the blue Monday, but by a blue Tuesday—the re-action from the labors of Sabbath and delaying for forty-eight hours. Likewise the phenomena of indigestion sometimes follow at once after a meal; in other cases not until several hours.—*Independent Practitoner.*

**Sciatica of Rheumatic Origin.**—I now show you another patient, who also complains of pain in the hip, thigh, and leg. His name is Charles M., and he is a barber by trade. He has had more or less of this pain for two years. Two years ago he had a severe attack of rheumatism, during which the large joints of the left leg swelled badly. Since that time he has suffered from pain in the left leg. He says that at the present he suffers most pain when standing. As you saw, when I pressed at the exit of the superior gluteal nerve on the left side, he jumped away. He also winces when I press on the posterior part of the thigh and in the popliteal space. There is, besides, tenderness in the calf of the leg. Inquiring into this man's general health, I find it to be fair; the tongue is clean, the appetite good, and the bowels move daily.

This history and these symptoms point very positively to sciatica or inflammation to the nerve sheath. You observe the man gives us a most unmistakable rheumatic history. He has almost constant pain, which is increased by standing. He has had the pain since having the attack of rheumatism to which he referred. It is needless to go farther into the history, for such symptoms make the diagnosis a positive one. The rheumatic history and character of pain leave us no doubt as to correctness of diagnosis.

To treat a case of this kind, if there were no chronic underlying cause, it would be proper to resort to hypodermic injections of either morphia or spirits of chloroform. In fact, there is no contra-indication for using either of them, as it is. We must not, however, be misled and overlook the fact that we have here a chronic cause, which is to be reached before we can expect to cure. I shall first advise that this man favor his limbs all he can. The muscles involved need absolute rest for some time, in order to bring the desired relief. In addition to the necessary rest I will order the following internal remedies:

℞ Potassii iodidi.................................. ʒ iiss.
Vini colchici rad................................ gtt. xlviij.
Syrupi zingiberis.
Aquæ........................................ aa f℥ ij. M
S. One dessertspoonful to be taken four times daily.

Chief among the great number of internal agents advised for muscular rheumatism are iodide of potassium, chloride of ammonium, and wine of colchicum root. If the patient be debilitated or anæmic, then recourse can be had to the tincture of iron, quinine, and arsenic.—*Col. and Clin. Rec.*

**On a New Method of Arresting Gonorrhea.**—I read with great pleasure the article headed as above by Mr. Cheyne, and wish to state that I have adopted his method of passing medicated bougies up the urethra for acute and chronic gonorrhea. The bougies I used were made by Kirby & Co., 14 Newman Street, Oxford Street. The other day I thought I would use iodoform in the shape of a bougie. I, therefore, ordered some containing five grains in each, and have been very gratified with the result, which has quite come up to my expectation. I have been in the habit of using iodoform, both in the form of ointment and of powder, for some years, and with marked success, in the treatment of indolent varicose ulcer of the leg, soft chancres, etc.

The method I adopt in the treatment of gonorrhea is this: I first order the patient an injection containing ten minims of liquor plumbi and two grains of sulphate of zinc to an ounce of water, to be used frequently until the acute symptoms have subsided. I then pass a No. 9 bougie up the urethra as far as the ulcerated spot. I then apply a piece of lint over the orifice of the urethra, under the prepuce, and tell him not to pass his urine for some hours afterward. I order him to take as little liquid as possible and no stimulants. I generally pass one or two bougies a day. My patients generally get rid of the gonorrhea in a week. The only constitutional treatment I adopt is a brisk purgative, followed by tonics.—J. B. James, M. R. C. S., in *British Med. Journal.*

[In the above we find confirmation of the idea given in a recent number of the *Southern Medical Record*, in which Dr. Word reports a case of gonorrhea cured by the use of an ointment of vaseline and bismuth introduced upon a bougie.—ED.]

**Milk and Lime Water.**—Milk and lime water are frequently prescribed by physicians in cases of dyspepsia and weakness of the stomach, and in some cases are said to be very beneficial. Many persons who think good bread and milk a great luxury, frequently hesitate to eat it for the reason that the milk will not digest readily: sourness of stomach will often follow. But experience proves that lime water and milk are not only food and medicine at an early period of life, but also at a later, when, as in the case of infants, the functions of digestion and assimilation are feeble and easily perverted. A stomach taxed by gluttony, irritated by improper food, inflamed by alcohol, enfeebled by disease, or otherwise unfitted for its duties—as is shown by the various symptoms attendant upon indigestion, dyspepsia, diarrhœa, dysentery, and fever—will resume its work, and do it energetically, on an exclusive diet of bread and milk and lime water. A goblet of cow's milk may have four tablespoonfuls of lime water added to it with good effect.—*The Druggist.*

**Strychnia Successfully Antidoted by Hydrate of Chloral.**—A case is reported, in the British Medical Journal, by George Gray, M.D., of strychnia poison successfully antidoted by chloral hydrate. The patient bought 20 grains of strychnia to poison rats with. He stirred it in a glass of whisky and drank it all. Dr. Gray administered 2 drachms of chloral hydrate in solution, with much difficulty, but it had the desired effect. Two days afterwards he was at his usual work.—*N. C. Med. Journal.*

**Iodoform in Otorrhœa.**—Chronic catarrh of the middle ear is notoriously obstinate in its course, yielding to no treatment ordinarily resorted to by the average practitioner of medicine. Having been disappointed in the results of treatment, even the manœuvres of Politzer's bag; inflating the drum cavity at regular intervals; systematic catheterizing and vaporizing with iodine; dilating the Eustachian tube; and all the internal medication usually employed—I was recently impressed with the idea of trying iodoform locally, and am surprised with the good results. Cases rebellious to everything usually done in such conditions have improved rapidly.

The following is my mode of treatment:

With a cotton carrier or any convenient instrument, and fine clean cotton wool, thoroughly cleanse the external auditory canal, down to the membrana tympani, using of course, delicateness of touch, so as to render no pain or reflex irritation of the upper air passage, causing cough, etc. Then apply the following powder every three days, or oftener if the case requires it, *i. e.*, if there is copious discharge of the offensive pus:

℞ Iodoform.......................................... ʒij

Tannic acid........................................... ʒj

Triturate very thoroughly, to an impalpable powder, and place a few grains of it in the end of an annealed glass tube about six inches long and ¼ of an inch in diameter. Then, with the thumb and forefinger of the left hand, pull the auricle upward and backward, thereby staightening the external auditory canal, and insert the loaded end of the annealed tube therein, apply the mouth of the other end of the tube, and give a gentle puff, throwing a whirlwind of medicinal dust down the passage, through the opening in the drumhead, if there be one, and there usually is in these cases, back into the mastoid cells, down the Eustachian tube, and completely storming the whole mucous lining of the auditory apparatus, and in a better manner than can be effected in any other way.—Dr. S. Pollock, in *Med. and Surg. Rep.*

**A New Anthelmintic.**—Dr. Lemos, in *Medicinische Neuigkeiten*, states that the ocinum basilicum, a plant known in Buenos Ayres under the name "albochaca," has an action of such a nature that the worms in every stage of development rapidly leave their location after the juice reaches them. Its use is so much the more to be recommended, since, if no worms should be present no injurious effect results from the plant, but a laxative and disinfectant action is the only result. Fifty grammes of the juice are given, followed in two hours by a dose of castor oil. A free discharge of the worms may be expected.—*Med. and Sur. Rep.*

**Metaphosphoric Acid.**—W. C. Grigg pronounces metaphosphoric acid a much more delicate test for albumen than nitric acid. The acid should be freshly made and dissolved without heat. A piece of the size of a pea is to be dissolved in a drachm of distilled water. The urine may be added to the solution or *vice versa.* If there be a trace of albumen the urine will immediately become turbid and of a milky-white color.—*Brit. Med. Journal.*

**Nutritive Enemata.**—Dr. Michelacci contributes an article on the subject of nutritive enemata, in *La Sperimentale*, in which he lays down the following general propositions. It is important to prepare the large intestine for the reception of nutrition by a previous injection of some simple substance. Whatever be the nature of the nutritive enema used, it should never be at a lower temperature than 37° or 38° Cent. (98.6° or 100.4° Fahr.).

It is, as a rule, preferable to give a considerable number of small enemata rather than a few large ones. The injection should be administered slowly and cautiously, and conveyed as high up as possible, by means of a long tube.

Each nutritive enema should be preceded by one of about two ounces of water, containing from four to eight drops of laudanum, in order to render the intestine more tolerant. In a clyster, alcohol should never be joined with peptone, as they are chemically incompatible. Fatty substances should be used sparingly, as they are difficult of absorption, and irritate the mucous membrane. Neutral or slightly alkaline enemata are frequently useful.

In cases in which symptoms of irritation or enteritis supervene, the author recommends suspending the use of all further enemata, or if this be not possible, then using opium freely, and selecting for injection only the less irritating substances.—*Med. and Surg. Reporter.*

**Benzoic Acid in Rheumatism.**—Senator has employed this remedy in forty-six cases of polyarticular rheumatism. From a clinical point of view it much resembles salicylic acid. Salkowsky attributes to it the same anti-putrescent and stronger anti-fermentive properties. In order not to irritate the primæ viæ, moderate doses are to be given at first: 10 to 12 grams (2½ to 3 drachms) of benzoic acid; or better 12 to 15 grams (3 to 3¾ drachms) of benzoate of soda. The acid was given as a powder in wafers or capsules; the benzoate in 100 to 140 grams (3 to 4 oz.) of an aromatic draught, with or without syrup.

It may be considered a specific for rheumatism, though in a less degree than salicylic acid. As a rule, the latter is more prompt in its action, though in some cases where that failed the benzoic acid succeeded.

No relapses nor cardiac complications have been observed. Benzoic acid seems to have no tendency to irritate the stomach or bowels. —*La Presse Medicale Belge.*

**Iron on Digestion.**—Dr. Perry, taking as his subject the Misuse of Iron Preparations, and their Effects upon the Digestive Process, supports his views with the following experiments. Two test-tubes containing artificial gastric juice and albumen; into one of them is poured one drachm of elixir of iron and quinia, at the end of four hours digestion has been completed in the tube containing no iron; the albumen remains unaffected in the tube containing the medicine. At the end of ten hours the albumen in the latter is still undigested, showing the effect of the iron preparation in suspending digestion. He quotes cases in which he considers indigestion to have been due to the presence of a preparation of iron in the stomach; he advises that such medicines be administered one hour before, or four hours after meals.—*Buff. Med. and Surg. Journal.*

**A New Mode of Distinguishing Central from Peripheral Paralysis of the Face.**—It is well known that this diagnosis can be readily made as a general thing by the constant current; the muscles in the paralyzed region contracting if the disease be central, and failing to respond if the nerve trunk itself be involved.

Dr. Strauss says that the diagnosis can be made just as readily by the use of jaborandi. If the disease be central, sweating occurs on both sides of the face, but if the nerve trunk be diseased, there is no sweating on the paralyzed side. He says this action is just as pathognomonic as that of electricity.

That the secretion of sweat is under the control of the nervous system, has now been positively established, and this observation of Strauss seems to show, as was to be expected, that the nerves governing the secretion of sweat in the face accompany the seventh pair.—*Va. Med. Monthly.*

**Saving Time.**—Dr. Clemenceau, the eminent French physician and member of the legislature, is remarkable for his quickness in the dispatch of business. Two men entered his consulting-room simultaneously the other day. The first, in reply to, "What is the matter?" said he had trouble in the chest, and was ordered to take off his shirt.

While prescribing the Doctor ordered the other visitor in, and said: "Just take your shirt off too; it will save time."

He immediately did so, and by the time the Doctor had written the prescription for the first man and received his fee he was stripped to the waist.

"You are suffering from pain in the chest, too, are you not?"

"Well, no," said patient No. 2, "I come to beg you would recommend me for a place in the post-office."

**A Substitute for Quinine.**—Dr. F. F. Habercorn urgently recommends the use of ethereal oil of mustard in fever, as being even superior to quinine. He has tried it in a great many cases, among others in more than fifty cases of chronic fever [intermittent?] He gives at a dose five drops of a ten-per-cent ethereal solution of oil of mustard, with two drops of an ethereal solution of thymol in a tablespoonful of water or wine; and in inveterate cases he adds three drops olei bassarum juniperi; six doses a day are given. While taking the medicine the patient must hold his breath.—*Louisville Med. News.*

**Jaborandi in Mumps.**—Dr. Testa states, in *Il Morgagni*, that he has employed this remedy in the form of infusion in five cases, and draws from his practice the following conclusions: 1. Jaborandi is an efficient remedy for mumps. 2. The efficacy is explained by its hydragogue, and especially its sialagogue properties. 3. Administered early it will prevent the development of the affection. 4. It may prevent the metastases which are not infrequent.—*Med. and Sur. Rep.*

**Caution in Regard to Chrysophanic Acid.**—Physicians prescribing chrysophanic acid should warn their patients against the accident of introducing it into their eyes with their fingers. Dilatation of the pupil ensues, accompanied with intense inflammatory itching and burning, causing much pain for the time it lasts, though the inflammasion soon subsides.—*Boston Journal of Chemistry.*

